# Supplementary material for: Modeling of Intracellular Taurine Levels Associated with Ovarian Cancer Reveals Activation of p53, ERK, mTOR and DNA-Damage-Sensing-Dependent Cell Protection
Source: Nutrients. 2024 Jun 9;16(12):1816. doi: 10.3390/nu16121816 (PMC11206249; doi:10.3390/nu16121816)
Supplement: Supplementary file 1 [file nutrients-16-01816-s001.zip › Nutrients supplemental figures.pdf]

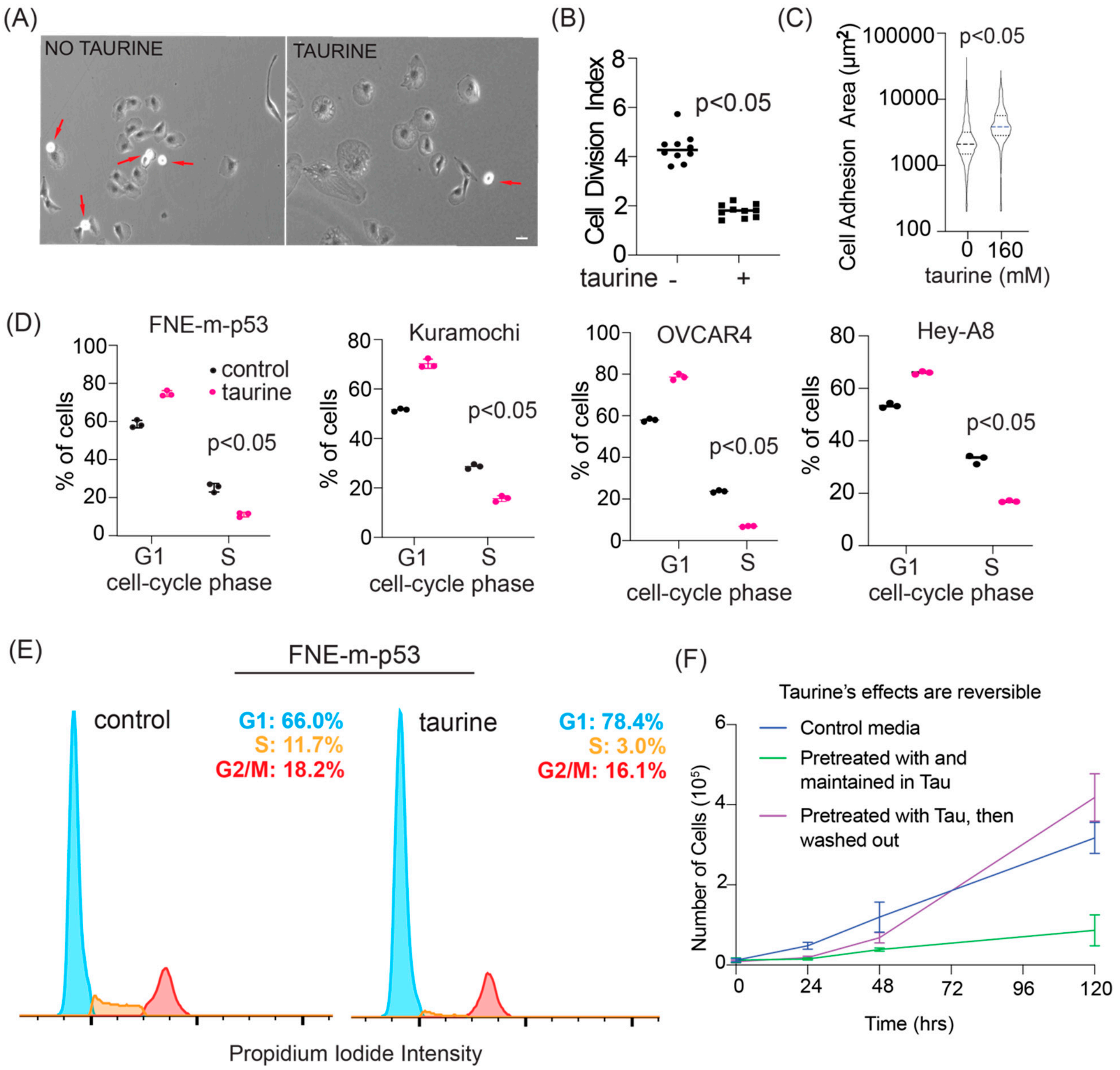

Supplemental Figure 1. (A) Phase-contrast images of FNE-m-p53 cells. The red arrows point to mitotic cells. Scale bar is 50 µm. (B) Quantification of FNE-m-p53 cell division based on live-cell fluorescent imaging. Cells were tracked for 72h using TrackMate. Cell division index is calculated as the number of tracks (cells) detected over time divided by the number of track splits (cell divisions). Each dot represents the mean index of one ROI. Statistical analysis was performed using student's t-test. (C) Quantification of GFP-expressing FNE-m-p53 cell adhesion area based on GFP signal obtained from live-cell microcopy. For the control group, n=2994 cells, and for taurine treatment, n=1164 cells. Statistical analysis was performed using student's t-test with Welch's correction. (D) Flow cytometry and PI-based cell-cycle analysis of cell lines treated with 160 mM taurine for 72h. Cell Cycle function in FlowJo was used to determine the frequency each cell-cycle phase. Each dot is one replicate. Bars indicate SD. Student's t-test was used to determine statistical significance. (E) Representative histograms for FNE-m-p53 cells shown in (D). (F) Quantification of cell proliferation based on automated counting. Cells were treated with control media

or media with taurine for three days. Cells were then replated, and taurine-treated cells were either washed and placed in control media or maintained in taurine for the indicated time. Cells were counted in triplicate.

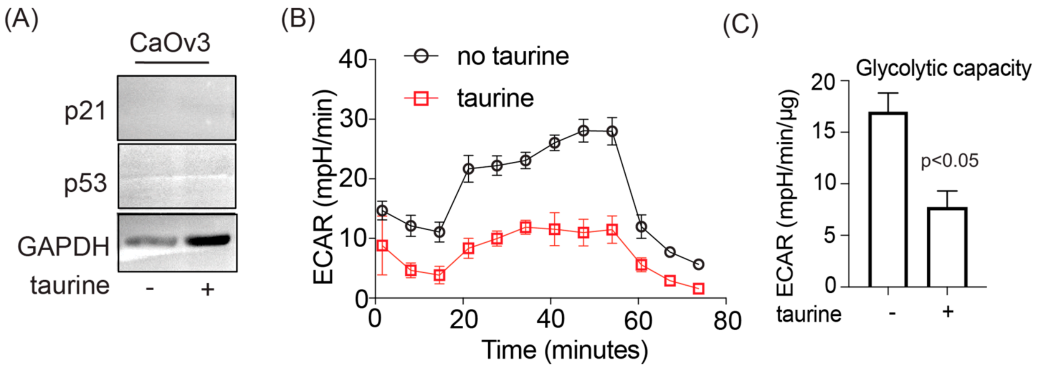

Supplemental Figure 2. (A) Western blot for p21 and p53 in CaOv3 cell monolayers treated with 160 mM taurine for 72h. (B) Analysis of ECAR over time representing glycolysis and (C) glycolytic capacity in TYK-nu cell monolayers treated with 160mM taurine. Bar graph shows the average values with standard deviations (maximal whiskers) across three experiments. An unpaired, two-tailed t-test was used to determine statistical differences between the means.

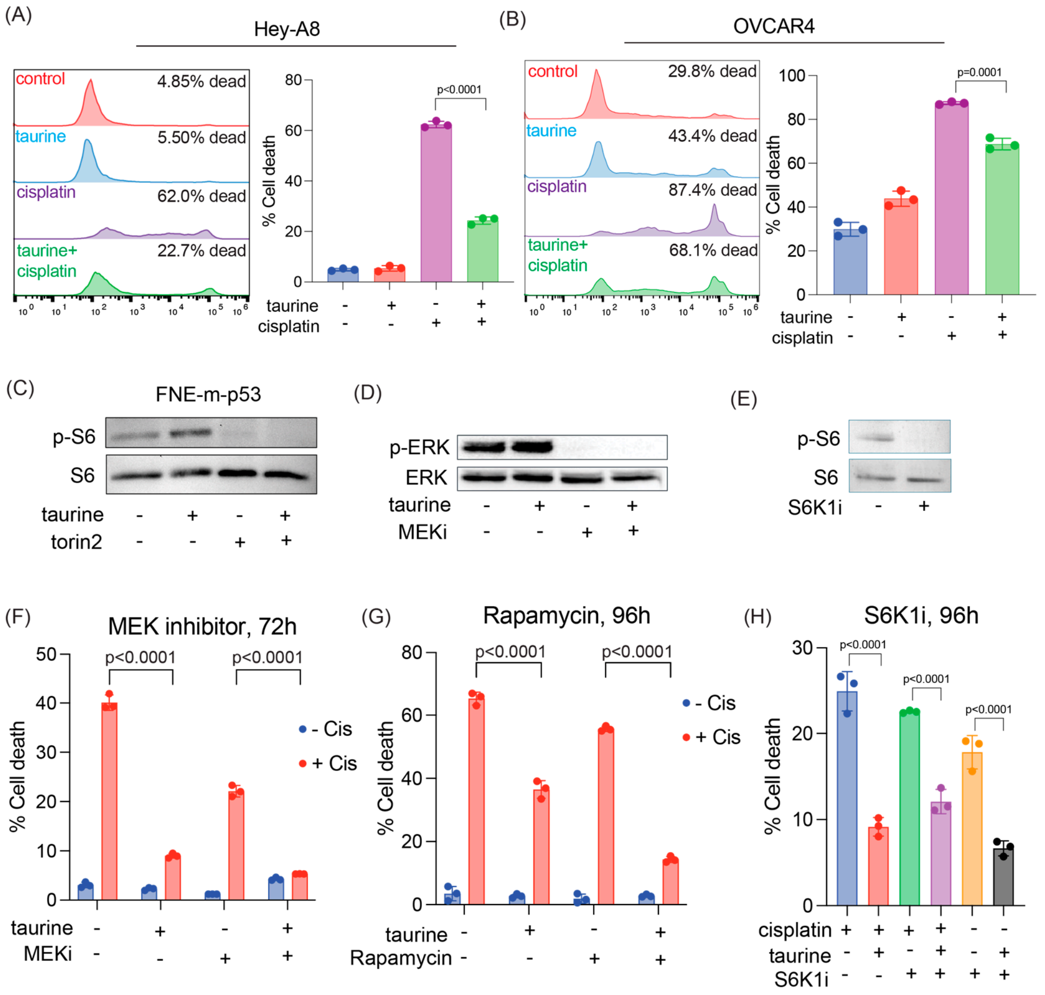

Supplemental Figure 3. (A-B) Representative histograms and quantification of PI incorporation of Hey-A8 (A) and OVCAR4 (B) cells treated under the indicated conditions for 72h. Statistical significance was determined by two-way ANOVA. p-values represent cisplatin vs. taurine + cisplatin treated groups. Each dot is one replicate. (C-E) Western blot of FNE-m-p53 treated with Torin2 (C), MEK inhibitor (D) or S6K1 inhibitor (E). (F-H) Quantification of PI incorporation in FNE-m-p53 cells treated with a MEK inhibitor (F), rapamycin (G), or S6K1 inhibitor under the indicated conditions. Each dot is one replicate. Statistical significance was determined by ANOVA. For all bar graphs, data are presented as mean ± SD.
